# Identification and rapid mapping of a gene conferring broad-spectrum late blight resistance in the diploid potato species *Solanum verrucosum* through DNA capture technologies

**DOI:** 10.1007/s00122-018-3078-6

**Published:** 2018-03-20

**Authors:** Xinwei Chen, Dominika Lewandowska, Miles R. Armstrong, Katie Baker, Tze-Yin Lim, Micha Bayer, Brian Harrower, Karen McLean, Florian Jupe, Kamil Witek, Alison K. Lees, Jonathan D. Jones, Glenn J. Bryan, Ingo Hein

**Affiliations:** 1 The James Hutton Institute, CMS, Errol Road, Dundee, DD2 5DA UK; 2 Present Address: Synpromics, Edinburgh, EH25 9RG UK; 3 Present Address: Columbia University, New York, NY 10027 USA; 4 The James Hutton Institute, ICS, Dundee, DD2 5DA UK; 5 Present Address: Monsanto, Saint Louis, 63167 USA; 6 The Sainsbury Laboratory, Norwich Research Park, Norwich, NR4 7GJ UK; 7 Scotland’s Rural College (SRUC), Peter Wilson Building, West Mains Road, Edinburgh, EH9 3JG UK; 8 School of Life Sciences, Division of Plant Sciences, University of Dundee at the James Hutton Institute, Dundee, DD2 5DA UK

## Abstract

**Key message:**

A broad-spectrum late blight disease-resistance gene from *Solanum verrucosum* has been mapped to potato chromosome 9. The gene is distinct from previously identified-resistance genes.

**Abstract:**

We have identified and characterised a broad-spectrum resistance to *Phytophthora infestans* from the wild Mexican species *Solanum verrucosum*. Diagnostic resistance gene enrichment (dRenSeq) revealed that the resistance is not conferred by previously identified nucleotide-binding, leucine-rich repeat genes. Utilising the sequenced potato genome as a reference, two complementary enrichment strategies that target resistance genes (RenSeq) and single/low-copy number genes (Generic-mapping enrichment Sequencing; GenSeq), respectively, were deployed for the rapid, SNP-based mapping of the resistance through bulked-segregant analysis. Both approaches independently positioned the resistance, referred to as *Rpi*-*ver1*, to the distal end of potato chromosome 9. Stringent post-enrichment read filtering identified a total of 64 informative SNPs that corresponded to the expected ratio for significant polymorphisms in the parents as well as the bulks. Of these, 61 SNPs are located on potato chromosome 9 and reside within 27 individual genes, which in the sequenced potato clone DM locate to positions 45.9 to 60.9 Mb. RenSeq- and GenSeq-derived SNPs within the target region were converted into allele-specific PCR-based KASP markers and further defined the position of the resistance to a 4.3 Mb interval at the bottom end of chromosome 9 between positions 52.62–56.98 Mb.

**Electronic supplementary material:**

The online version of this article (10.1007/s00122-018-3078-6) contains supplementary material, which is available to authorized users.

## Introduction

Potato is the most important non-cereal food crop and consumed by more than a billion people worldwide (Birch et al. 2012). The oomycete pathogen *Phytophthora infestans* causes late blight disease of potato and led to the Irish famine in the mid-1840s. Despite significant breeding efforts, potato late blight disease continues to represent the most serious threat to potato production due to the considerable adaptability of the pathogen (Haas et al. 2009). A conservative estimate of the chemical control costs and yield losses associated with late blight exceeds €5.2 billion annually (Haverkort et al. 2009). Efforts to control late blight disease by harnessing naturally occurring resistances from wild potato species led, in part, to the establishment of global potato germplasm collections that are now systematically screened for novel resistance (*R*) genes (Vossen et al. 2014; Van Weymers et al. 2016).

Mapping and cloning of *R* genes belonging to the nucleotide-binding and leucine-rich repeat (NB-LRR) family is aided by a detailed knowledge concerning their genomic organisation. In plant genomes, *R* genes are often found within physical clusters, which are thought to be an important feature of their evolution (Michelmore and Meyers 1998). The organisation of potato *R* genes has been studied following the release of the doubled monoploid *Solanum tuberosum* group Phureja clone DM1-3 516 R44 (DM) genome (PGSC 2011; Jupe et al. 2012). The *R* gene annotation was further improved through targeted enrichment sequencing of NB-LRR genes (RenSeq), which identified over 750 NB-LRR sequences (Jupe et al. 2013). Furthermore, RenSeq technology has proven effective in the mapping of novel resistances (Jupe et al. 2013) and the identification of candidates in combination with long-read enrichment sequencing (Giolai et al. 2016; Witek et al. 2016). RenSeq can also serve as a diagnostic tool (dRenSeq) utilising high-stringent post-enrichment sequence mapping conditions against a customised reference set consisting of known functional genes. dRenSeq enables a rapid and massively parallel sequence comparison to ascertain whether the underlying *R* gene is based on already characterised NB-LRRs or if it is a novel resistance (Van Weymers et al. 2016; Jiang et al. 2018).

The number of cloned functional NB-LRR genes from *Solanum* species that are effective against late blight continues to grow (Rodewald and Trognitz 2013; Vossen et al. 2016; Witek et al. 2016). Included in this list are *Ph3* from *S. pimpinellifolium* (Zhang et al. 2014), *Rpi*-*vnt1* from *Solanum venturii* (Pel et al. 2009; Foster et al. 2009), *R8* (Vossen et al. 2016) and *R9a/Rpi*-*edn2* (Jo et al. 2015) from *S. demissum* and *Solanum x edinense* that have all been identified on the lower end of chromosome 9 and which is known to contain NB-LRR-rich regions (Jupe et al. 2013).

Here, we report the characterisation and mapping of a novel late blight resistance from a *S. verrucosum* accession (Ver54) through two complementary target enrichment sequencing strategies, RenSeq (Jupe et al. 2013) and Generic-mapping enrichment sequencing of single/low-copy number genes (GenSeq). Both approaches yielded SNPs that are linked to the resistance at the distal end of potato chromosome 9. RenSeq-derived reads from resistant parent Ver54 and F_1_ clone Ver95/8a6 were also used in a dRenSeq analysis to ascertain if the resistance was novel.

## Materials and methods

### Potato material

Screening of the Commonwealth Potato Collection (CPC) for late blight resistance identified *Solanum verrucosum* (*S. verrucosum*) accession 54 as highly resistant and *S. verrucosum* accession 3939 as very susceptible. Resistant accession 54, clone number 15 (hereafter referred to as Ver54) was crossed to susceptible accession 3939 clone 17 (hereafter referred to as Ver3939). Progeny from the resulting F_1_ population were all resistant and the F_1_ clone Ver95/8a6 was backcrossed to susceptible parent Ver3939 to give rise to backcross population (BC_1_) Ver96/40 that contained 152 clones.

### Late blight assessment

Late blight testing was conducted through whole plant assays, seedling tests, and detached leaf experiments using potato cultivar Craig’s Royal as a susceptible control. Whole plant and seedling resistances were assessed according to Bradshaw et al. (2006) and Stewart et al. (1983), respectively. Detached leaf tests were carried out as described previously by Whisson et al. (2007). Disease was scored when symptoms were established in susceptible control plants (potato cultivar Craig’s Royal) between 5 and 8 days post-infection. The severity of infection was recorded on a scale ranging from 1 (very susceptible) to 5 (very resistant) for seedling and detached leaf tests and for whole plants according to the Malcolmson scale (Cruickshank et al. 1982), where 1 represents very susceptible to 9—very resistant with no symptoms. A minimum of two independent replicates per late blight assessment was conducted.

### RenSeq and GenSeq enrichment and read mapping

All plant DNA extractions were performed using the DNeasy Plant Mini kit (QIAGEN) according to the manufacturer’s protocol. RenSeq and GenSeq target enrichment sequencing were both conducted according to Van Weymers et al. (2016) using genomic DNA and identical protocols (Fig. S1). The bait sequences for RenSeq (version 3; Jupe et al. 2013) and GenSeq can be accessed at http://solanum.hutton.ac.uk. Post-enrichment, individually indexed parent resistant, parent susceptible, bulked resistant, and bulked susceptible samples were sequenced alongside a further eight samples with paired-end (2 × 250 bp) Illumina MiSeq chemistry in a single flow cell. Reads were first quality and adapter trimmed with fastq-mcf (v1.04.676; Aronesty 2011) to a minimum base quality of 20 as detailed in Van Weymers et al. (2016). All reads have been submitted to the European Nucleotide Archive (https://www.ebi.ac.uk/ena) with the ENA Accession Number PRJEB23360 (https://www.ebi.ac.uk/ena/data/view/PRJEB23360).

For dRenSeq analysis, the RenSeq reads from Ver54, F_1_ clone Ver95/8a6 and Ver3939 were trimmed using cutadapt 1.9.1 (Martin 2011) and mapped against a customised reference set of 16 known NB-LRR genes using Bowtie2 (v2.2.1; Langmead and Salzberg 2012) in very-sensitive default mode. Discordant mappings were disabled, and up to ten valid mapping positions were reported per read pair with an alignment score cutoff of 5 for a 250 bp read pair. The reference set used was based on Van Weymers et al. (2016) and included *R1* (GenBank: AF447489.1; Ballvora et al. 2002), *R2* (GenBank: FJ536325.1; Lokossou et al. 2009), *R2*-*like* (GenBank: FJ536323.1; Lokossou et al. 2009), *R3a* (GenBank: AY849382.1; Huang et al. 2005), *R3b* (GenBank: JF900492.1; Li et al. 2011), *Rpi*-*sto1* (GenBank: EU884421.1; Vleeshouwers et al. 2008), *Rpi*-*pta1* (GenBank: EU884422.1; Vleeshouwers et al. 2008), *Rpi*-*blb1* (GenBank: AY426259.1; van der Vossen et al. 2003), *Rpi*-*blb2* (GenBank: DQ122125.1; van der Vossen et al. 2005), *Rpi*-*blb3* (GenBank: FJ536346.1; Lokossou et al. 2010), *Rpi*-*abpt* (GenBank: FJ536324.1; Lokossou et al. 2009), *Rpi*-*vnt1.1*, and *Rpi*-*vnt1.3* (GenBank: FJ423044.1; Foster et al. 2009) and three additional genes *Rpi*-*amr3* (GenBank:KT373889; Witek et al. 2016), *R8* (GenBank: KU530153; Vossen et al. 2016), and *R9a/Rpi*-*edn2* (Jo et al. 2015; https://www.google.com/patents/US20140041072).

For the genetic mapping of the resistance through RenSeq or GenSeq, the trimmed RenSeq- or GenSeq-derived reads were mapped to the potato DM reference genome (v4.03; PGSC 2011; Sharma et al. 2013) using Bowtie2 (v2.0.6; Langmead and Salzberg 2012) in very-sensitive default mode. Discordant and mixed mappings were disabled, and all other parameters were set to the default value. The BAM files for the bulks were merged and indexed using SAMtools (v0.1.18; Li et al. 2009), as were the BAM files for the parents. Pileup files were generated for the bulks and parents using SAMtools mpileup and piped into VarScan (v2.3.7; Koboldt et al. 2012) for variant calling.

### SNP filtering

SNPs were filtered using custom Java code to retain only informative SNPs. The analysis was solely focused on bi-allelic SNPs. For the resistant bulk, each progeny clone was presumed to be heterozygous at the resistance locus, which should yield an overall frequency of 50% for the resistance allele. Consequently, a threshold of 40–60% alternate allele was set to identify SNPs that are linked to the resistance. In contrast, for the susceptible bulk, all diploid individual members are presumed to be devoid of the resistant allele (0%) and the overall frequency of the susceptible allele in the bulk is expected to be close to 100%. A threshold of < 10 or > 90% alternate allele was set to identify the SNPs that are linked to the resistance gene depending on the phasing of the reference sequence (Fig. S1). Similarly, for the parents enriched via RenSeq, the threshold was set to 40–60% alternate allele in the resistant parent (heterozygous F_1_ clone Ver95/8a6), with the susceptible parent Ver3939 exhibiting the opposite allele (again with < 10 or > 90% alternate allele cutoff depending on the phasing). For the homozygous parents Ver54 and Ver3939 enriched via GenSeq, the threshold was set to < 10 or > 90% alternate allele, respectively. The minimum read depth was set to 50. BEDTools intersect (v2.20.1; Quinlan and Hall 2010) was used to relate the SNP locations to genes based on the PGSC v3.4 gene annotations for GenSeq (Table S1) and *R* gene annotations for RenSeq (Jupe et al. 2013).

### KASP assay development

We used enrichment sequencing-based SNPs residing between genes DMG400010287 and DMG400017146 on Chromosome 9 to develop Competitive Allele-Specific PCR (KASP) markers (Table S2). Flanking sequences (50 bp each upstream and downstream) around the SNP positions were used to design KASP primers (KASP by Design Oligos, LGC Genomics limited). Initially, the performance and accuracy of all KASP assays were validated by testing the markers on three genotypes including homozygous-resistant clone Ver54, homozygous-susceptible clone Ver3939, and heterozygous-resistant clone VER95/8a6. As a result, 12 informative KASP assays successfully reproduced all expected SNP genotypes. This set of KASP markers was used for genotyping individual plants from the resistant and susceptible bulks. One additional KASP marker, representing a sequence polymorphism in DMG400017237, was also included in the analysis (Table S2).

The KASP reaction mix was prepared for a total reaction volume of 8.11 µl, which contained 4 µl of genomic DNA (5 ng/µl), 4 µl of 2xKASP Master Mix (LGC Genomics limited), and 0.11 µl of KASP assay mix (KASP by Design Oligos, LGC Genomics limited). PCR was performed on StepOnePlus using the following thermal cycling program: 2 min at 20 °C (Pre PCR read); 15 min at 94 °C (initial activation); ten touchdown cycles of 20 s at 94  °C and 1 min at 62 °C (decreasing by 0.7 °C  per cycle); and finally 32 cycles at 94 °C for 20 s followed by 55 °C for 1 min. Post-PCR read was performed at 20  °C for 2 min. The SNP genotype was determined using StepOne Software v2.3 (Life Technologies).

## Results

### *Rpi*-*ver1* is a broad-spectrum, dominant-resistance gene

**Fig. 1:**
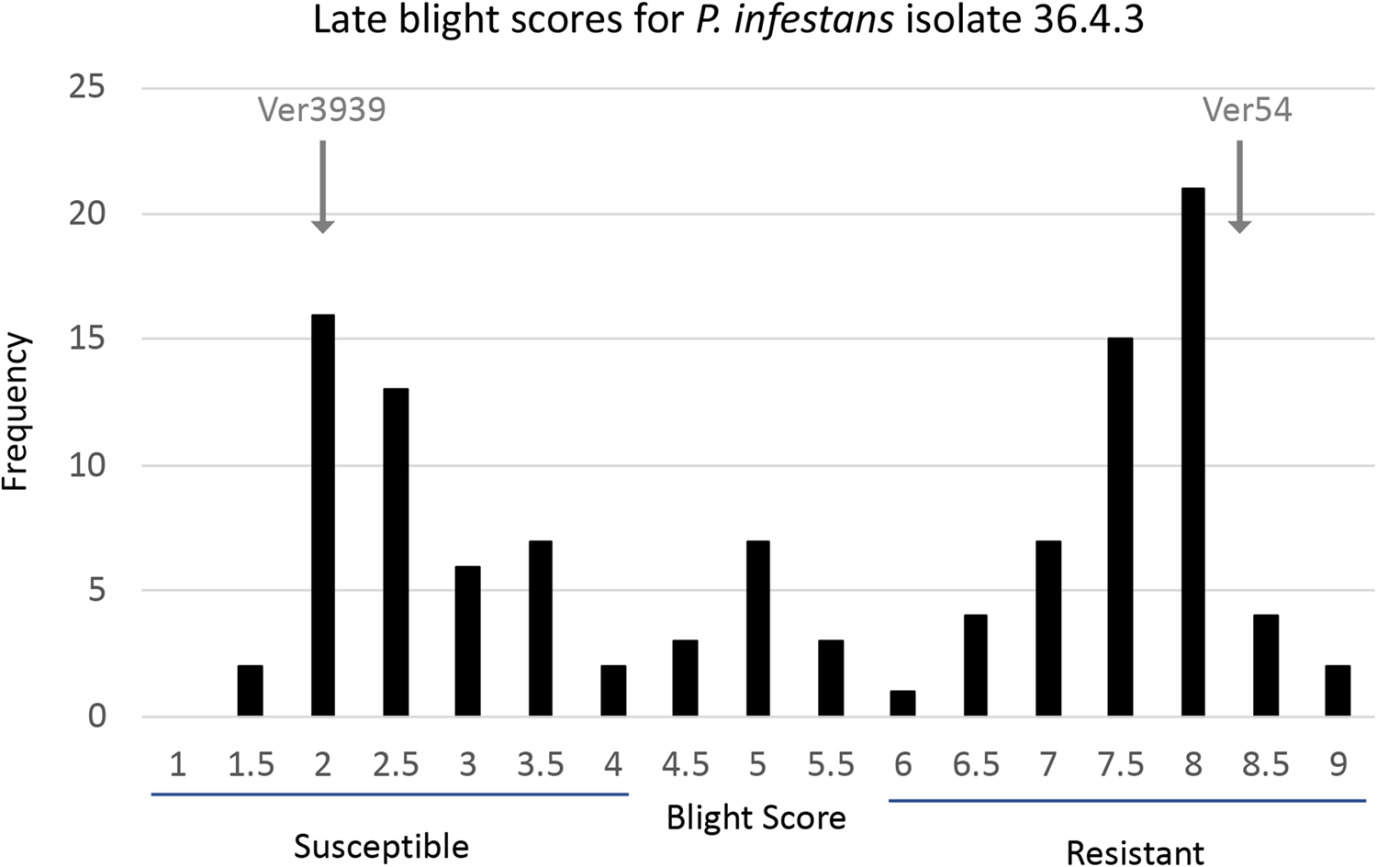
Blight scores for 113 clones from backcross population VER96/40 assessed with *P. infestans* isolate 36.4.3 in at least two replicates. The 1–9 scale represents the spectrum of responses from totally susceptible (1) to absolute resistant (9). Indicated are the phenotypic scores of the parents and the plants determined to be resistant or susceptible, respectively

Screening wild potato species from the Commonwealth Potato Collection (CPC) for late blight resistance with *Phytophthora infestans* isolate 36.4.3 (race 1,2,3,4,6,7) in whole plant tests identified resistant *S. verrucosum* accession Ver54. In repeated whole plant blight screenings, resistant clone Ver54 scored 8.3 on a 1–9 scale of resistance (1 susceptible–9 resistant) and was crossed to susceptible clone Ver3939 that scored 2.0 in independent screens. Clones from the resulting F_1_ population were all resistant and F_1_ clone Ver95/8a6 that scored an average of 8.2 in independent tests was backcrossed to susceptible clone Ver3939 to give rise to backcross population (BC_1_) Ver96/40. Whole plant replicated blight tests using the same isolate were carried out on 113 clones of the BC_1_ population. Of the individual clones tested, 54 were unambiguously classified as resistant with a score of ≥ 6.0 and 46 as susceptible with a score of ≤ 4.0 (Fig. 1). A Chi-square test confirmed that resistance and susceptibility segregation fits into a 1:1 ratio (*x*^2^ = 0.64, *p *> 0.43), suggesting that the resistance is controlled by a single dominant gene.

To investigate the broadness of the resistance, Ver54 was further challenged with diverse pathogen isolates through whole plant tests. Each independent assessment included at least three plants and utilized the isolates CP (race 1, 3, 4, 7, 10, 11), LC1 (race 1, 2, 4, 10, 11), 37.1.1 (1, 2, 3, 4, 6, 7), SASA 01/29 (1, 2, 3, 4, 6, 7, 8, 10, 11), and 07/39 (1, 2, 3, 4, 5, 6, 7, 9, 10, 11). In all cases, Ver54 scored between 8 and 9. The contemporary isolate 07/39 belongs to an A2 mating type known as genotype 13_A2, which is also commonly referred to as ‘blue13’ and is highly aggressive (Cooke et al. 2012). Because of the emerging importance of this pathogen genotype for potato production, 76 clones of the BC_1_ population Ver96/40 and parental lines Ver95/8a6 as well as Ver3939 were assessed for resistance towards *P. infestans* isolate 07/39 in independent whole plant tests. The two parents showed distinct phenotypes with blight scores of 8.0 and 2.3, respectively. The results for independently validated, high confidence individual clones were qualitatively similar to the blight test conducted with *P. infestans* isolate 36.4.3 and allowed us to identify progeny that consistently displayed resistance (with a score of ≥ 6.0) and susceptibility (with a score of ≤ 4.0) towards both isolates for a bulked-segregant analysis approach (Table S3).

### dRenSeq reveals the resistance to be distinct from known resistances

**Fig. 2:**
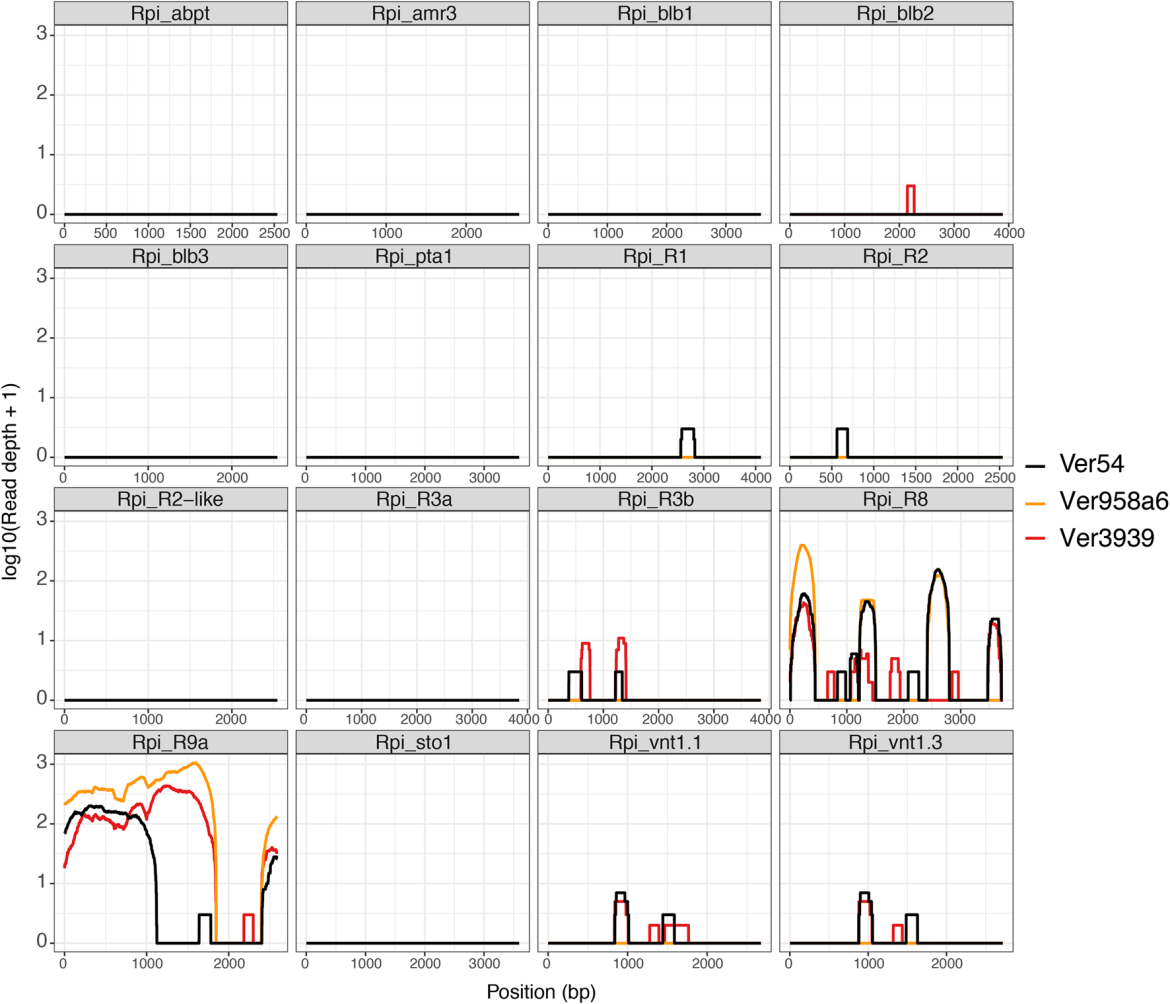
dRenSeq analysis on resistant plants Ver54, resistant F_1_ clone Ver95/8a6 and susceptible plant Ver3939. RenSeq-derived reads are mapped against a customised reference set of 16 known NB-LRR genes in very-sensitive mode. Each box represents an entire reference gene from the start codon to the stop codon (*x*-axis) and the *y*-axis reveals the coverage of the genes by RenSeq-derived reads on a log scale

Ver54, Ver95/8a6, and Ver3939 were found to be unsuitable for large-scale effectoromics screens (Vleeshouwers et al. 2011a) as the plants often produced non-specific responses to different *Agrobacterium tumefaciens* strains as well as to PVX toothpick inoculations (results not shown). Instead, to establish if the resistance in *S. verrucosum* 54 is novel or based on an already characterised resistance gene, a dRenSeq analysis was conducted according to Van Weymers et al. (2016). Genomic DNA of resistant parent Ver54, resistant F_1_ clone Ver95/8a6, and susceptible parent Ver3939 was enriched using NB-LRR-specific probes (Jupe et al. 2013). Post-enrichment RenSeq reads were mapped, at high stringency, against 16 functional late blight NB-LRR genes. The analysis revealed that none of the reference NB-LRR genes were fully represented by RenSeq reads. This provides evidence that the Ver54 resistance is, on a nucleotide level, distinct from all known NB-LRRs (Fig. 2). We hereafter refer to the new resistance as *Rpi*-*ver1*.

### RenSeq mapping places the *Rpi*-*ver1* on chromosome 9

Owing to the success of bulked-segregant NB-LRR gene enrichment and sequencing through RenSeq (Jupe et al. 2013) and the possibility that *Rpi*-*ver1* could be a member of the NB-LRR gene family, RenSeq-based mapping was conducted. Genomic DNA libraries were prepared from the susceptible parental clone Ver3939 and the F_1_ resistant clone Ver95/8a6. Included in the enrichment were also resistant and susceptible bulks derived from BC_1_ population Ver96/40. The bulks were selected based on the independently validated phenotypic data of blight resistances to isolates 36.4.3 and 07/39 (Table S3). Bulked resistance (BR) contained 19 individual clones with an average disease score of ≥ 7.2 to both isolates and bulked susceptible (BS) 21 clones that scored ≤ 2.4 in the combined tests. The bulks contained equal amounts of DNA from each of the clones selected. The bulks, as well as the clones Ver95/8a6, and Ver3939 were individually indexed prior to enrichment and paired-end sequencing using Illumina MiSeq (Jupe et al. 2013).

Following RenSeq, the percentage of reads on target was calculated as the number of reads mapping to an annotated, targeted RenSeq region in the DM reference genome (Van Weymers et al. 2016). For the RenSeq analysis, more than 76% of reads could be mapped to the DM genome and the percentage of reads mapping to NB-LRR targets varied between 57.81 and 64.27% (Table 1), which is indicative of a successful enrichment considering that NB-LRRs account for less than 1% of the entire potato genome (Jupe et al. 2012, 2013).

**Table 1:** Paired-end MiSeq read analysis after target enrichment

Enrichment	Sample		Reads mapped to DM		Reads on target	
			No.	(%)	No.	(%)
RenSeq	Bulks	Resistant (R/S)	2,929,498	76.28	1,788,205	61.04
		Susceptible (S/S)	3,080,868	77.42	1,781,038	57.81
	Parents	VER95/8a6 (R/S)	4,819,088	76.05	3,097,390	64.27
		Ver3939 (S/S)	3,467,942	77.24	2,197,678	63.37
GenSeq	Bulks	Resistant (R/S)	2,430,952	79.38	1,473,689	60.62
		Susceptible (S/S)	3,340,570	79.37	2,026,228	60.66
	Parents	Ver54 (R/R)	4,598,124	78.35	2,574,524	55.99
		Ver3939 (S/S)	4,791,574	79.22	2,730,604	56.99

The number of reads and the percentage mapped to the potato reference genome DM are shown alongside the number of reads and % mapped on NB-LRR target. The target region contains the template for the enrichment bait library design + 1000 bp of flanking sequence up- and downstream. RenSeq utilized previously identified NB-LRR sequences as targets (Jupe et al. 2013) and GenSeq COS markers alongside other conserved or low-copy number genes

SNPs were filtered to retain only informative SNPs that conformed to the specific ratios expected for the monogenic nature of the resistance (Fig. S1). In total, 102 SNPs were identified in the bulks and, with the exception of chromosomes 2 and 7, located to all chromosomes including chromosome 0 that contains currently unanchored contigs. Similarly, 1355 SNPs were identified between the heterozygous-resistant clone Ver95/8a6 and the susceptible parent Ver3939 and located to all chromosomes apart from chromosome 7 (Table 2). However, when combined, and selected for SNPs that could only be unambiguously identified for both parents and the bulks, 26 SNPs passed these filter criteria. Of these SNPs, one located to a single gene on chromosome 11 (PGSC0003DMG402030235) and the remaining 25 to 7 annotated NB-LRR genes located on chromosome 9. The relative position of these genes in DM is shown in Table 3 and Fig. S2.

**Table 2:** SNP analysis of RenSeq reads

Chromosome	Number of SNPs			Number of RenSeq genes with SNPs
	Bulks	Parents VER95/8a6 (R/S) Ver3939 (S/S)	In bulks and parents	
1	4	6	0	0
2	0	22	0	0
3	2	11	0	0
4	7	84	0	0
5	3	329	0	0
6	2	4	0	0
7	0	0	0	0
8	3	23	0	0
9	41	165	25	7
10	7	112	0	0
11	7	490	1	1
12	12	50	0	0
0	14	59	0	0
Total	102	1355	26	8

The number of filtered SNPs identified from VarScan that conform to the expected SNP ratio for the bulks, parents, and both is shown. The SNPs and the underlying NB-LRR genes are organised by potato chromosomes 1–12 and currently unknown positions (chromosome 0)

**Table 3:** Positional mapping of RenSeq SNPs

Chromosome	Gene ID	Start [bp]	End [bp]
9	RDC0001NLR0215	49,173,508	49,176,574
9	PGSC0003DMG400010287	49,910,733	49,917,963
9	PGSC0003DMG400017146	56,983,606	56,993,036
9	RDC0001NLR0223	59,673,213	59,678,764
9	RDC0001NLR0226	59,697,054	59,701,335
9	PGSC0003DMG400031521	60,086,974	60,090,937
9	RDC0001NLR0230	60,956,586	60,959,599
11	PGSC0003DMG402030235	44,214,788	44,219,141

The gene identifier and chromosomal position of NB-LRR genes that contain filtered SNPs that conform to the expected SNP ratio for the bulks and parents are shown. The start and end positions of the genes (in base pairs) on the respective chromosome are shown

### GenSeq enrichment rapidly identifies genic flanking markers for the resistance

To complement and confirm the RenSeq data, which can only reveal linkage within the proximity of known *R* gene loci, we conducted enrichment sequencing for 1980 low-copy number genes that are anchored to the potato genome, and which include 1163 conserved orthologous sequence (COS) genes (Table S1). A customised bait library was used that tiled the targeted genes. The approach was termed generic-mapping enrichment sequencing (GenSeq) to distinguish it from RenSeq. The same indexed samples that were generated for RenSeq analysis were subjected to GenSeq enrichment sequencing with the exception of the resistant parent, which in the case of GenSeq was homozygous Ver54 instead of heterozygous Ver95/8a6 used for RenSeq.

Following GenSeq, the percentage of reads on target was calculated as the number of reads mapping to an annotated, targeted GenSeq region in the DM genome (Table S1; Table 1). The calculations were in line with those conducted for RenSeq detailed above and revealed similar mapping and on-target rates. More than 78% of reads could be mapped to the DM genome and the percentage of reads on target varied between 55.99 and 60.66% (Table 1).

SNPs were filtered as detailed above. When combined and selected for SNPs that could only be unambiguously identified for both parents and the bulks, 38 SNPs passed these filter conditions and corresponded to 22 individual genes (Table 4). Of these genes, 20 located to chromosome 9, and one each to chromosomes 2 and 5, respectively (Tables 4 and 5). The relative position of these genes is shown in Table 5 and Fig. S3.

**Table 4:** SNP analysis of GenSeq reads

Chromosome	Number of SNPs			Number of GenSeq genes with SNP
	Bulks	Parents VER54 (R/R) Ver3939 (S/S)	In bulks and parents	
1	5	252	0	0
2	6	367	1	1
3	7	483	0	0
4	6	239	0	0
5	4	358	1	1
6	3	415	0	0
7	6	588	0	0
8	5	317	0	0
9	52	204	36	20
10	6	409	0	0
11	2	462	0	0
12	1	191	0	0
0	5	37	0	0
Total	108	4322	38	22

The number of filtered SNPs identified from VarScan that conform to the expected SNP ratio for the bulks, parents and both is shown. The SNPs and the underlying COS and low-copy number genes are organised by potato chromosomes 1–12 and currently unknown positions (chromosome 0)

**Table 5:** Positional mapping of GenSeq SNPs

Chromosome	Gene ID	Start [bp]	End [bp]
2	PGSC0003DMG400015491	20,799,347	20,808,028
5	PGSC0003DMG400030559	3,812,966	3,817,781
9	PGSC0003DMG400007716	45,923,714	45,932,545
9	PGSC0003DMG400000954	46,684,825	46,688,571
9	PGSC0003DMG400012882	47,540,375	47,541,519
9	PGSC0003DMG400012878	47,707,264	47,709,221
9	PGSC0003DMG400019345	48,245,208	48,251,906
9	PGSC0003DMG400031427	49,658,888	49,663,619
9	PGSC0003DMG400010295	50,119,632	50,127,918
9	PGSC0003DMG400003803	51,128,968	51,130,104
9	PGSC0003DMG400003805	51,176,158	51,182,923
9	PGSC0003DMG400016850	51,715,245	51,718,154
9	PGSC0003DMG400011361	52,173,667	52,179,993
9	PGSC0003DMG401011368	52,406,217	52,411,550
9	PGSC0003DMG400011395	52,448,497	52,453,430
9	PGSC0003DMG400011375	52,572,167	52,574,502
9	PGSC0003DMG400011401	52,622,427	52,624,950
9	PGSC0003DMG400015117	55,566,611	55,573,561
9	PGSC0003DMG400017164	56,633,867	56,638,761
9	PGSC0003DMG400017161	56,740,433	56,743,667
9	PGSC0003DMG400001506	57,162,917	57,172,504
9	PGSC0003DMG400026427	59,225,168	59,230,126

The gene identifier and chromosomal position of genes re-sequenced through GenSeq that contain filtered SNPs that conform to the expected SNP ratio for the bulks and parents are shown. The start and end positions of the genes (in base pairs) on the respective chromosome are shown

### Genotyping of segregating populations with converted KASP makers refines the *Rpi*-*ver1* map position to a 4 Mb interval

Based on RenSeq- and GenSeq-derived SNPs (Tables 2, 3, 4 and 5), 12 KASP markers (Table S2) were successfully designed for chromosome 9 to represent polymorphisms identified in four resistance genes (NLR0215, DMG400017146, NLR0226, and DMG400031521) and eight GenSeq sequences (DMG400012878, DMG400019345, DMG400031427, DMG400010295, DMG400003805, DMG400016850, DMG400011361, and DMG400011401). These markers were used to genotype the individuals used in the resistant and susceptible bulks from the segregating BC_1_ population Ver96/40 alongside parental clones Ver54, Ver95/8a6, and Ver3939.

**Fig. 3:**
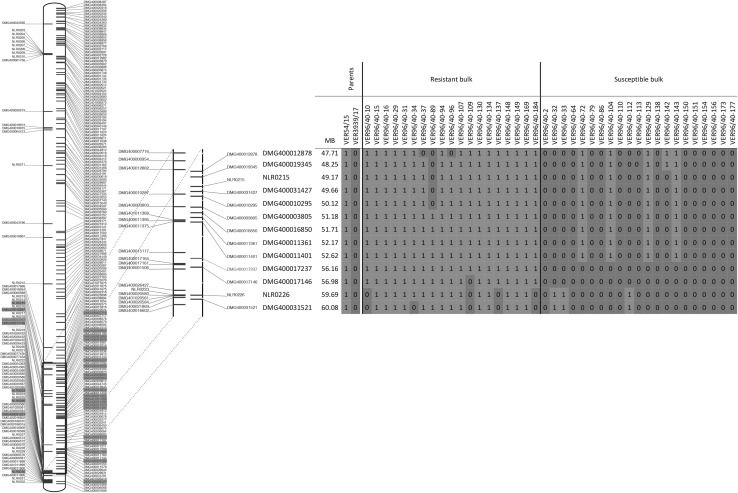
Graphical representation of the RenSeq and GenSeq mapping data on chromosome 9. Shown on the left is an overview of chromosome 9 with the positions and identities of resistance genes represented by RenSeq probes shown in green and single/low-copy number genes represented through GenSeq probes in blue. Highlighted in yellow are RenSeq or GenSeq represented genes that show polymorphisms associated with the resistance. A close-up of the interval with significant polymorphism is shown in the middle and genes for which we designed KASP markers are positioned on the right side. The additional KASP marker developed for DMG400017237 that co-segregates with the resistance is shown in red. The position of these genes, based on DM, is shown in Mb (mega-bases). The graphical genotyping results are shown on the right. The resistance genotype as found in Ver54 (homozygous) is represented with a green ‘1’ and the susceptible genotype associated with Ver3939 (homozygous) is represented as a blue ‘0’. The genotypes of 19 resistant and 21 susceptible plants, used in the bulked-segregant analysis, are shown. Recombination points are identifiable for when the genotypes alternate between green resistance allele (1) and blue susceptible allele (0) and vice versa (colour figure online)

Initial analysis of the recombination events enabled narrowing of the *Rpi*-*ver1* resistance locus to approximately 4.3 Mb based on the potato reference genome from DM between GenSeq marker DMG400011401 [52.62 Mb] and RenSeq marker DMG400017146 [56.98 Mb]. The mapping data represented through graphical genotyping (Fig. 3) revealed that *Rpi*-*ver1* associates closely with the RenSeq-derived resistance gene marker DMG400017146 as only one recombinant was found amongst the bulked resistant sample and none in the bulked susceptible progeny (Fig. 3). Importantly, the physical order of RenSeq- and GenSeq derived KASPs, as informed by the DM reference genome, corresponded well to the genetic data as the number of recombinants increased towards the proximal and distal end of chromosome 9 the further we moved away from DMG400017146.

With this information in mind, and using DM as a reference, we selected one additional gene, DMG400017237, to develop an additional KASP marker in this genetically defined interval between GenSeq marker DMG400011401 and RenSeq-derived marker DMG400017146 (Table S2). In DM, DMG400017237 is predicted to reside at position 56.16 Mb on potato chromosome 9 and, therefore, less than 1 Mb from DMG17146. However, graphical genotyping revealed that this new marker co-segregated with the resistance in the parents and bulks (Fig. 3).

## Discussion

The potato species *S. verrucosum* is a wild, diploid, inbreeder within the tuber-bearing Solanum family. *S. verrucosum* originates from Mexico, and like many other Mexican potato species, has developed late blight resistance, presumably as a consequence of close co-evolution with the oomycete pathogen *P. infestans* (Hein et al. 2009, Vleeshouwers et al. 2011b). Indeed, an allele mining strategy in *S. verrucosum* has previously identified functional orthologs of *RB*, originally identified in another Mexican potato taxon, *S. bulbocastanum* (Song et al. 2003, Liu and Halterman 2006). In this study, we report on the identification, characterization and mapping of a novel, broad-spectrum late blight resistance gene, *Rpi*-*ver1*, in *S. verrucosum* 54 using the state-of-the-art enrichment sequencing.

*Rpi*-*ver1* maps to a region on the long arm of chromosome 9 where a number of resistance genes have previously been identified, characterised, and cloned, including *Rpi*-*vnt1* from *S. venturii*, (Pel et al. 2009, Foster et al. 2009), *Rpi*-*mcq1* from *S. mochiquense* (Smilde et al. 2005), and *Ph*-*3* conferring late blight resistance in tomato (Zhang et al. 2014). All three resistance genes are members of the same gene family showing high homology to Tm-2^2^ from *S. lycopersicum* (Foster et al. 2009). In addition, two late blight resistance genes from *S. demissum*, *R8,* and *R9a* have also been identified on the bottom part of chromosome 9 (Vossen et al. 2016 and Jo et al. 2015). The combination of *P. infestans* isolates used in this study (36.4.3, CP, LC1, 37.1.1., 36.4.3, SASA 01/29 and 07/39) was designed to be virulent on the R1–R11 resistance genes. Yet, the resistance in Ver54 remained functional against all of these isolates which suggests that R*pi*-*ver1* is functionally distinct from these genes.

Indeed, dRenSeq analysis (Van Weymers et al. 2016) confirmed that the resistance in Ver54 is distinct at the nucleotide level from 16 known potato resistances that include, for example, *Rpi*-*blb1* (*RB*), *Rpi*-*blb2,* and *Rpi*-*vnt1*.*1*/*Rpi*-*vnt1.3* (Fig. 2). The highest sequence representation of any reference *R* genes, which was nevertheless only partial, was observed for *R8* and *R9a*, which were already ruled out as the genes underlying the resistances based on late blight pathogen testing. DRenSeq analysis was critical in ascertaining the novelty of the resistance, as *S. verrucosum* is highly recalcitrant for high-throughput effectoromics screens that are typically based on *Agrobacterium*-based effector recognition studies following transient delivery including through *Agrobacterium*/PVX toothpick inoculations.

Importantly, dRenSeq is a cost-effective step to include in the search for novel resistances as the dRenSeq and RenSeq analyses use the same *R* gene enriched sequencing data from the parents of segregating populations with opposing phenotypes. We use RenSeq-based enrichment followed by dRenSeq analysis on parental material routinely now before committing to genetic screens and bulked-segregant analysis. In this study, we utilized Ver54 alongside the resistant F_1_ clone Ver95/8a6 and susceptible Ver3939 for dRenSeq (Fig. 2) and then generated and included RenSeq reads for the bulk resistant and bulk susceptible plants for the RenSeq-based mapping. The on-target rates of reads corresponding the NB-LRR-type sequences were between 57.81 and 64.27% which ensures sufficient coverage for dRenSeq and subsequent SNP analysis (Van Weymers et al. 2016; Jupe et al. 2013).

Another technical advance from this study was the utilization of GenSeq, a novel tool for mapping traits without any a priori knowledge about the type of genes that could be responsible for the phenotype. GenSeq is, therefore, more generic than RenSeq in facilitating the analysis of traits other than pest or disease resistance. However, in this study, GenSeq not only confirmed the RenSeq-based mapping position on potato chromosome 9, but helped develop markers that flank the resistance locus (Fig. 3). Importantly, the exact same genomic DNA libraries used for RenSeq-sequencing could be used for GenSeq, which saved on the costs for DNA extraction, shearing, indexing, and purification prior to enrichment. GenSeq is somewhat similar to exome capture (Teer and Mullikin 2010), but focuses the enrichment on gene coding DNA fragments that can be mapped to the potato genome with high confidence as the representative genes targeted for probe development are either single or low-copy number genes. Unlike other procedures that reduce the genome complexity prior to sequencing and mapping such as GBS or RAD (Andrews et al. 2016), the resolution of GenSeq is, however, restricted to the genes that were used for the bait design. Nevertheless, due to the enrichment step, an on-target rate of between 55.99 and 60.66% was achieved, which allowed us to filter for SNPs that were supported by at least 50 individual reads prior to filtering for significance.

Due to the stringent SNP filtering conditions applied to RenSeq and GenSeq bulked-segregant analysis, 96% (25 SNPs out of 26) and 94.7% (36 SNPs out of 38) of the identified SNPs, respectively, corresponded to the mapping position on chromosome 9, which was validated through KASP assays on individual plants. The KASP conversion rate itself had a higher than 80% success rate, which further supports the efficacy of our SNP calling post-enrichment sequencing.

Future work will focus on the fine mapping and the isolation of the gene responsible for the late blight resistance on chromosome 9. This work will be facilitated using an expanded population of over 1000 individuals and the development of additional markers. This activity will be supported by the availability of a draft genome assembly from *S. verrucosum* Ver54, which is currently being constructed (Paajanen et al. submitted).

### Author contribution statement

XC, DL BH, AL, and KM conducted the late blight screening and mapping. XC, FJ, and MA conducted the enrichment sequencing. KB, T-YL, MB, and MA conducted the computational analysis and KASP design. KM, FJ, and JJ contributed with GenSeq. IH, GB, and XC wrote the manuscript. IH designed the molecular experiments and secured funding.

## Electronic supplementary material

Below is the link to the electronic supplementary material.

Supplementary material 1 (TIFF 3835 kb) **Fig. S1:** Graphical overview of RenSeq, dRenSeq and GenSeq (top) and SNP filtering conditions for bulked resistant and bulked susceptible progeny based on mapping of post-enriched reads to the DM reference genome (bottom).

Supplementary material 2 (TIFF 896 kb) **Fig. S2:** Graphical representation of NB-LRRs that contain informative SNPs linked to *Rpi-ver1*. Chromosomes 1-12 are depicted on the x-axis and the numbers of genes with informative SNPs within a 1Mb interval are shown as dots on chromosome 9 (six genes) and chromosome 11 (one gene). Shaded in the background are the numbers of genes that were assessed at each locus and represent in this case the position of known NB-LRRs used for the bait library design.

Supplementary material 3 (TIFF 2193 kb) **Fig. S3:** Graphical representation of GenSeq sequences that contain informative SNPs linked to *Rpi-ver1*. Chromosomes 1-12 are depicted on the x-axis and the numbers of genes with informative SNPs within a 1Mb interval are shown as dots on chromosome 2 (one gene), chromosome 5 (one gene) and chromosome 9 (20 genes). Shaded in the background are the numbers of genes that were assessed at each locus and represent in this case the position of the COS and additional genes used for the bait library design.

Supplementary material 4 (XLSX 137 kb) **Table S1.** Single and low-copy number potato genes used for GenSeq enrichment library generation. Shown are the chromosome position, coordinates, gene IDs (PGSC0003DMG identifiers), and basic annotation information.

Supplementary material 5 (XLSX 9 kb) **Table S2.** KASP marker details. Shown are the gene IDs (PGSC0003DMG identifiers), relative position of the gene on potato chromosome 9 [Mb] as well as the nucleotide sequences surrounding the SNPs [resistance nucleotide / susceptible nucleotide].

Supplementary material 6 (XLSX 9 kb) **Table S3.** Late blight scores for clones from backcross population VER96/40 assessed with *P. infestans* isolate 36.4.3 and 07/39 in at least two independent replicates and used to generate bulked resistant (BR) and bulked susceptible (BS) samples. The 1–9 scale represents the spectrum of responses from totally susceptible (1) to absolute resistant (9).
