# Supplementary figures and images for: Identification and rapid mapping of a gene conferring broad-spectrum late blight resistance in the diploid potato species Solanum verrucosum through DNA capture technologies
Source: Theor Appl Genet. 2018 Mar 20;131(6):1287–97. doi: 10.1007/s00122-018-3078-6 (PMC5945768; doi:10.1007/s00122-018-3078-6)

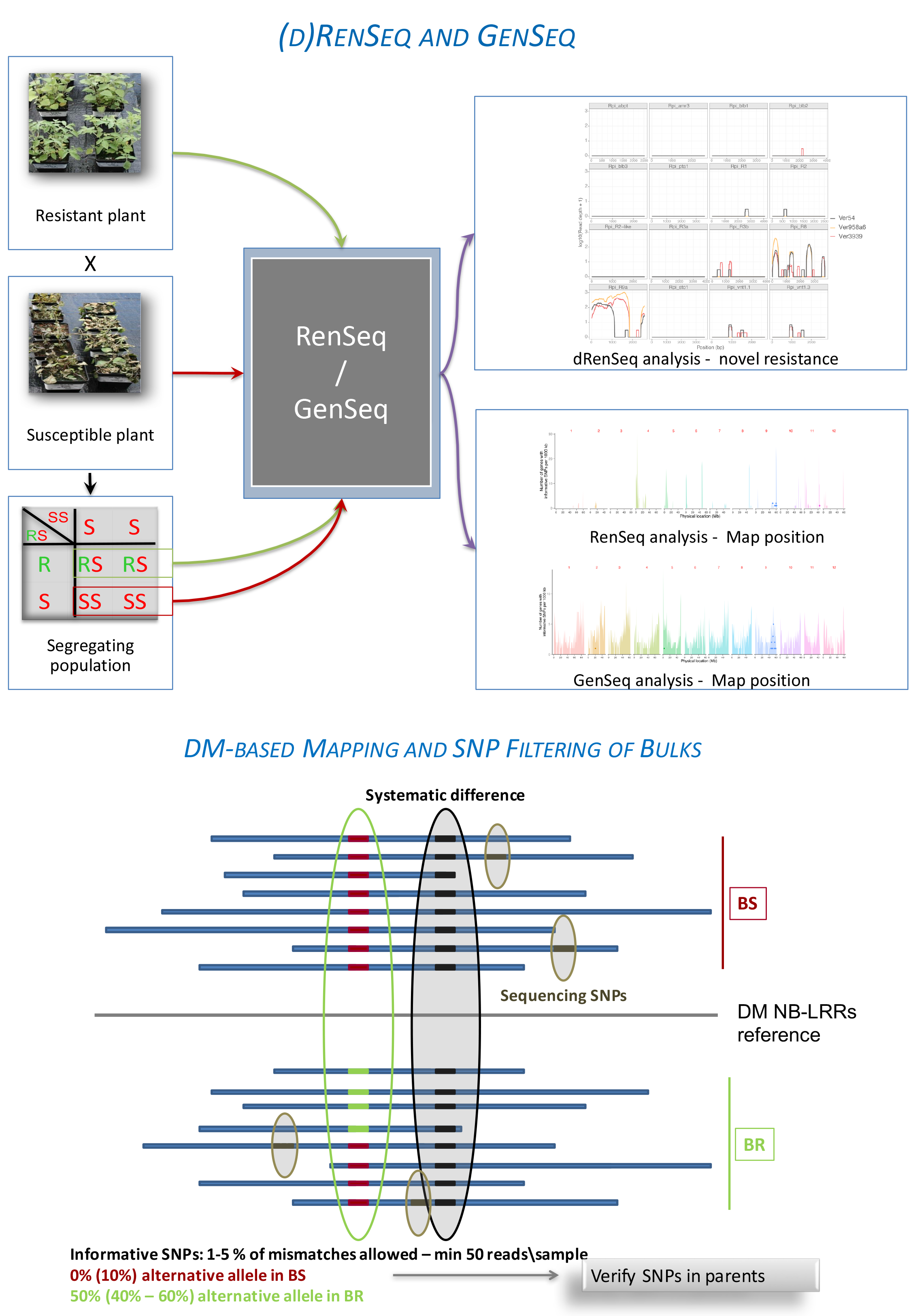

Supplement: Supplementary file 1 — Supplementary material 1 (TIFF 3835 kb) Fig. S1: Graphical overview of RenSeq, dRenSeq and GenSeq (top) and SNP filtering conditions for bulked resistant and bulked susceptible progeny based on mapping of post-enriched reads to the DM reference genome (bottom). [file 122_2018_3078_MOESM1_ESM.tif]

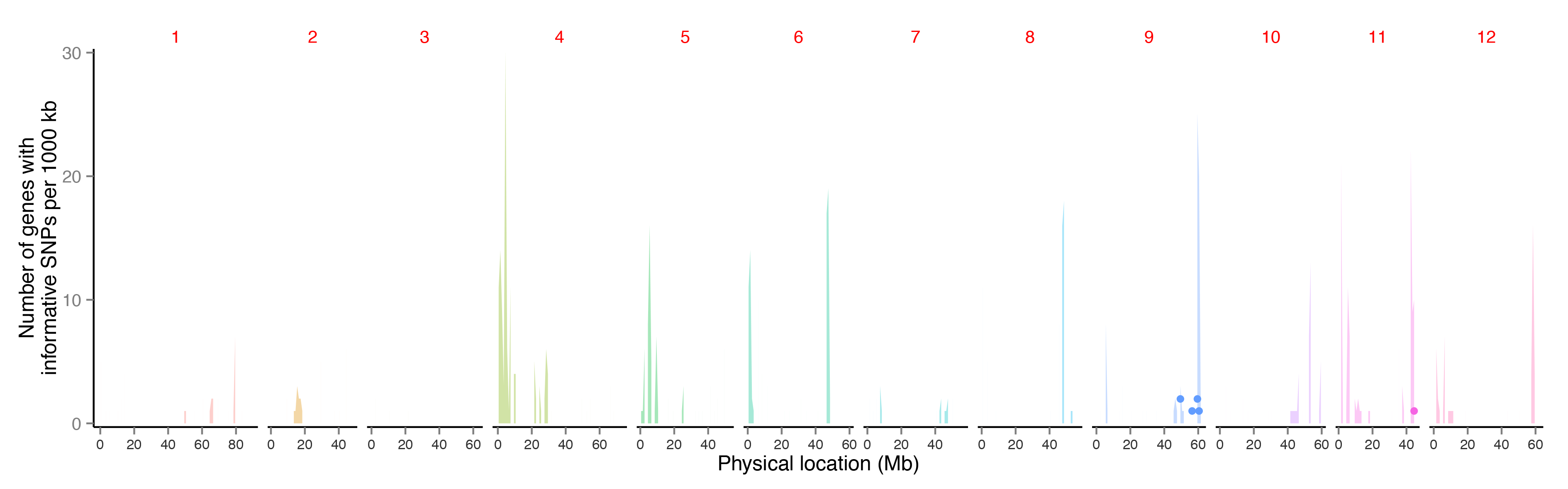

Supplement: Supplementary file 2 — Supplementary material 2 (TIFF 896 kb) Fig. S2: Graphical representation of NB-LRRs that contain informative SNPs linked to Rpi-ver1. Chromosomes 1-12 are depicted on the x-axis and the numbers of genes with informative SNPs within a 1Mb interval are shown as dots on chromosome 9 (six genes) and chromosome 11 (one gene). Shaded in the background are the numbers of genes that were assessed at each locus and represent in this case the position of known NB-LRRs used for the bait library design. [file 122_2018_3078_MOESM2_ESM.tif]

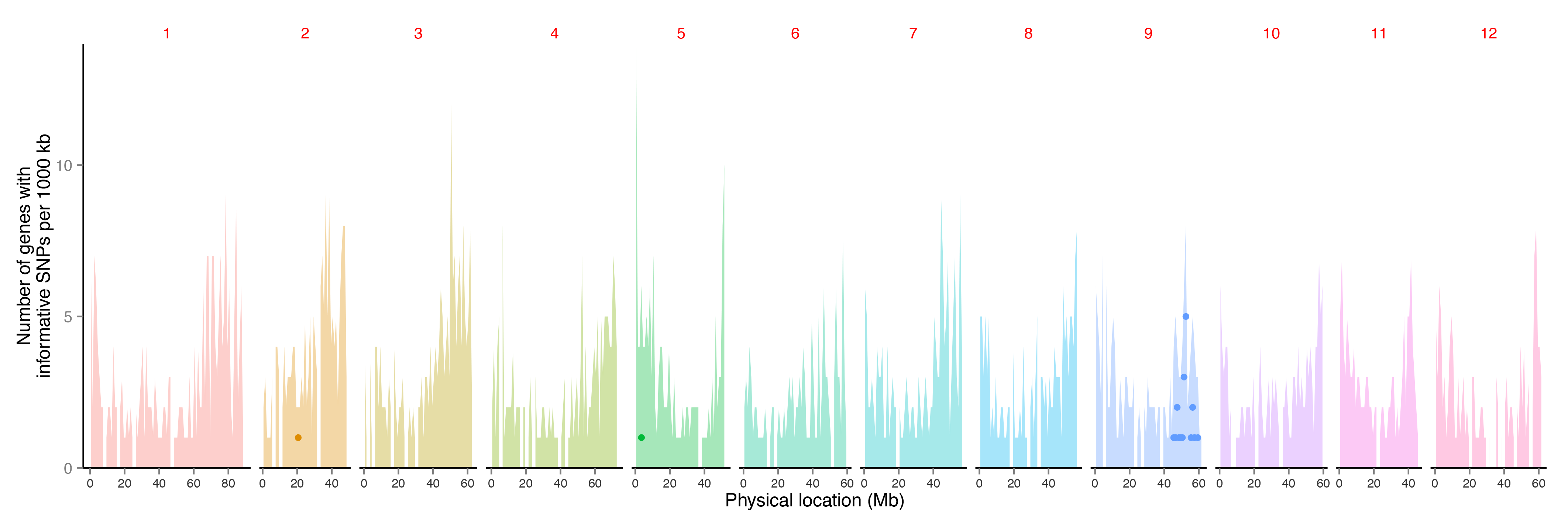

Supplement: Supplementary file 3 — Supplementary material 3 (TIFF 2193 kb) Fig. S3: Graphical representation of GenSeq sequences that contain informative SNPs linked to Rpi-ver1. Chromosomes 1-12 are depicted on the x-axis and the numbers of genes with informative SNPs within a 1Mb interval are shown as dots on chromosome 2 (one gene), chromosome 5 (one gene) and chromosome 9 (20 genes). Shaded in the background are the numbers of genes that were assessed at each locus and represent in this case the position of the COS and additional genes used for the bait library design. [file 122_2018_3078_MOESM3_ESM.tif]
